# Low back pain presentations to rural, regional, and metropolitan emergency departments

**DOI:** 10.1111/ajr.12854

**Published:** 2022-03-01

**Authors:** Simon R. E. Davidson, Steven J. Kamper, Robin Haskins, Michael O'Flynn, Karen Coss, John Paul Smiles, Amanda Tutty, Jane Linton, Joe Bryant, Maree Buchanan, Christopher M. Williams

**Affiliations:** ^1^ School of Medicine and Public Health University of Newcastle Newcastle New South Wales Australia; ^2^ 5260 Hunter New England Population Health Hunter New England Local Health District Wallsend New South Wales Australia; ^3^ School of Health Sciences University of Sydney Camperdown New South Wales Australia; ^4^ 223690 Nepean Blue Mountains Local Health District Penrith New South Wales Australia; ^5^ John Hunter Hospital Outpatient Services New Lambton Heights New South Wales Australia; ^6^ John Hunter Hospital Emergency Department New Lambton Heights New South Wales Australia; ^7^ Tamworth Hospital Emergency Department Tamworth New South Wales Australia; ^8^ Clarence Health Services Physiotherapy Department Northern New South Wales Local Health District Grafton New South Wales Australia; ^9^ Aboriginal Health Strategy Unit Coffs Harbour New South Wales Australia; ^10^ 5260 Health Analytics and Business Support Unit Hunter New England Local Health District New Lambton Heights New South Wales Australia

**Keywords:** musculoskeletal disorders, public hospital, regional health, rural health, service improvement

## Abstract

**Objective:**

To describe the context of low back pain (LBP) presentations to emergency departments (EDs) by remoteness areas, hospital delineation level and staffing portfolios.

**Design:**

A retrospective observational study using routinely captured ED and admission data over a 5‐year period (July 2014–June 2019).

**Settings:**

Thirty seven EDs across a large health district in NSW, Australia, covering major cities, inner regional areas and outer regional areas.

**Participants:**

Emergency department (ED) presentations with a principal or secondary diagnosis of LBP based on ICD‐10 code (M54.5).

**Main outcome measures:**

ED presentation and associated admission measures, including presentation rate, referral source, time in ED, re‐presentation rate, admission details and cost to the health system.

**Results:**

There were 26 509 ED presentations for LBP across the 5 years. Time spent in ED was 206 min for EDs in major cities, 146 min for inner regional EDs and 89 min for outer regional EDs. Re‐presentation rates were 6% in major cities, 8.8% in inner regional EDs and 11.8% in outer regional EDs. Admission rates were 20.4%, 15.8% and 18.8%, respectively.

**Conclusions:**

This study describes LBP presentations across 37 EDs, highlighting the potential burden these presentations place on hospitals. LBP presentations appear to follow different pathways depending on the ED remoteness area, delineation level and staff portfolio.

**What is already known on this subject:**

- Low back pain is the 5th most common reason for presentation to Australian emergency departments
- Low back pain presentations to emergency departments are well described in metropolitan areas
- Higher presentation rates for low back pain have been reported in rural areas compared with metropolitan areas

**What does this study adds?**

- There are considerable differences in presentations of patients with low back pain in different areas of remoteness, particularly related to time in ED, re‐presentation rate and admission rate
- Service‐level outcomes including time in ED and re‐presentation rate are different depending on ED staffing portfolio and inconsistent across hospital delineation level
- The study shows that strategies to improve care for patients with low back in the ED likely need to consider the local context at each facility. A one‐size‐fits‐all approach is unlikely to be effective

## 1 INTRODUCTION

Low back pain (LBP) is common and disabling and causes reduced quality of life.^1^, ^2^ Approximately 84% of individuals are likely to develop LBP at some time in their life.^3^ While international guidelines recommended initial management in primary care, many patients with LBP present to hospital emergency departments (EDs).^4^ LBP is the 5th most common cause for presentations to Australian EDs.^5^ Numerous Australian and international studies have described ED presentations for LBP,^6^, ^7^, ^8^, ^9^ but most concentrate on metropolitan hospitals.

Regional and rural hospitals face different operational challenges compared with metropolitan facilities. Clinician capabilities and available resources and services in EDs are often specific to the location and hospital delineation.^10^, ^11^ Time pressures for understaffed facilities in rural areas, limited opportunities for professional development related to musculoskeletal conditions, and limited access to Fellowship of the Australasian College of Emergency Medicine (FACEM)–trained physicians may lead to higher variation in the management of LBP. Metropolitan EDs are often staffed by FACEM physicians who are specifically trained to manage presentations such as LBP in the ED. In contrast, regional and rural EDs are staffed by General Practitioner Visiting Medical Officers (GPVMO). They also work as general practitioners in the community and therefore require a broader field of knowledge. These variations may account for differences in the management of LBP in EDs across these areas.^12^ Rural EDs also have more demand per capita and fewer options to support the management of patients upon discharge.^5^, ^13^ One previous study reported higher presentation rates for LBP in regional and rural areas than in metropolitan areas.^14^ Australian national data show residents of remote and very remote areas are more likely to have been to an ED in the past 12 months than their metropolitan counterparts due to limited general practitioner availability.^13^, ^15^ Access to medical specialists (e.g. orthopaedic or neurosurgical teams) is also poorer in rural areas.^13^, ^15^

The differing demand, hospital facilities and external medical services for EDs in regional and rural areas mean the challenges for managing LBP may not be the same as metro EDs. Despite this, rural and regional hospitals are often held to similar service‐level performance indicators. For example, all New South Wales (NSW) public hospital EDs are currently measured against the same performance indicators. The emergency treatment performance target is a statewide mandate that calls for ED processes to be completed within 4 h for most ED presentations.^16^ The planning of services and support mechanisms should consider ED performance indicators and the specific challenges that facilities face.

There has been no documentation of LBP presentations to EDs across different localities to guide service support for regional and rural EDs.^17^ This study aimed to describe characteristics of LBP presentations and service‐level performance indicators to EDs across different remoteness areas, hospital delineation levels and ED staffing portfolios.

## 2 METHODS

This multi‐centre retrospective observational study uses linked ED and admission data captured by electronic medical records for LBP presentations to 37 hospital EDs located in a large NSW Health District (Hunter New England Local Health District [HNELHD]) between 01/07/2014 and 30/06/2019. Ethics approval was granted by the HNELHD Human Research Ethics Committee (2019/ETH12178).

### 2.1 Sample

The health district is a large NSW public health service, encompassing 131 785 km^2^, with a total patient catchment area of 912 352 people in 2016.^18^, ^19^ The Australian Statistical Geography Standard Remoteness Structure defines five categories of remoteness: major cities, inner regional areas, outer regional areas, remote areas and very remote areas.^20^, ^21^ Approximately 51% of the health district population is classified as residing in a major city area, 35% in an inner regional area, and 14% in outer regional and remote areas.^22^

### 2.2 Data capture

The Emergency Department Data Collection, which forms part of the Health Information Exchange, contains records of all ED presentations across NSW Health jurisdictions. The data originate from the Patient Administration System, which tracks the patient from their arrival, through their ED visit (+/‐ hospital admission), and includes basic discharge information. Upon discharge from ED, the treating clinician enters a principal diagnosis code (+/− a secondary diagnosis code), which documents the reason for the ED presentation. If the patient is admitted, coders review the clinical notes and determine the diagnosis codes to assign. Available diagnostic codes are based on a subset of the International Statistical Classification of Diseases and Related Health Problems, 10th Revision (ICD‐10) codes.^23^

We identified and extracted data from the health information exchange for all presentations to the EDs within the health district for LBP within the study period based on a principal or secondary diagnosis code of LBP (M54.5). An independent data analyst confirmed the code captured relevant LBP presentations and omitted specific causes (e.g. fracture, spinal cord injury, cauda equina syndrome and radicular low back pain). To ensure no relevant presentations were missed, we completed a keyword search of all ED presentations using the keywords of ‘back’ AND ‘pain’ but NOT ‘back of’. We queried admitted patient data to capture information from any admissions (inpatient wards and emergency short‐stay units) directly following ED presentation for the patients identified with LBP through ED records.

We extracted data for the variables described in Table 1.

**TABLE 1:** Variables extracted

**ED presentation data**
Demographic data (age, gender, postcode, Indigenous status, private health insurance status),
Presenting problem description and code
Presentation date/time
Triage code^a^
Date/time 1st seen by clinician
Discharge date/time
Facility name
ED referral source and ED visit type
Mode of discharge from ED
Principal diagnosis code and description
National Weighted Activity Unit (NWAU) type
NWAU value
NWAU version
Re‐presentation within 5 days (Yes/No)
Hours to re‐presentation (where relevant)
**Admission data (where relevant)**
Admission date/time
Discharge date/time
Total length of stay (LOS) for the acute admission
Total NWAU for the acute admission
NWAU version
Australian refined diagnosis‐related group (DRG) description and code
Last acute ward, speciality code and speciality name during admission

^a^ Triage codes use the Canadian Triage and Acuity Scale (CTAS), which is a 5‐point scale where patients are scored depending on their acuity of illness; it ranges from resuscitation (immediately life‐threatening condition) to non‐urgent (chronic or minor condition).^39^, ^40^

We coded arrival time as AM or PM, and arrival in or out of business hours (business hours 09:00–17:00). We calculated time in ED using presentation and discharge dates/times. We calculated the cost of presentations using the National Weight Activity Unit (NWAU) for each presentation or admission, multiplied by the national efficient price (NEP) for the relevant NWAU version. We based NWAU figures on all relevant variables for the year of ED presentation and associated admission (e.g. for ED presentation, Indigenous status, compensable status, funding eligibility indicator, establishment remoteness area and Urgency Disposition Group [UDG] code).^24^ To maintain a homogeneous sample, presentations were limited to 12 years of age and older. We based this decision on consensus from the investigator group considering the rising prevalence of non‐specific low back pain from age 12.^25^, ^26^, ^27^ An audit of presentations for patients less than 12 years revealed that most were trauma‐related or incorrectly coded.

### 2.3 Analysis

We summarised descriptive data using mean (standard deviation [SD]) or median (inter‐quartile range [IQR]) for continuous variables, depending on distribution, and *n* (%) for categorical variables.

We used three hospital classification methods to describe the facilities.

1. Hospital postcode to categorise according to remoteness. There were 5 EDs in a major city, 13 in an inner regional area and 19 in an outer regional area. There are no facilities in remote or very remote areas within the health district.
2. Delineation levels to categorise according to hospital service capabilities. All EDs in NSW are classified according to a delineation level, which describes minimum workforce and service requirements for safe clinical service delivery.^28^, ^29^ Delineation level also indicates the care available to patients (e.g. specialist input, imaging availability and allied health availability) and allows comparison with facilities in other NSW areas. There is 1 Level 6 facility within the health district, and 2 Level 5, 3 Level 4, 8 Level 3, 16 Level 2 and 7 Level 1 facilities.^29^
3. Staffing portfolio; either specific ED staff (FACEM) or GPVMOs. Within the health district, 7 facilities are staffed by FACEM staff and 29 by GPVMO. One facility has a shared model of care involving FACEM and GPVMO.

## 3 RESULTS

### 3.1 Sample

There were 26 509 ED presentations for LBP by 22 042 individuals between July 2014 and June 2019. Of the 37 EDs in the health district (198 beds), 36 facilities had ED presentations for LBP during the 5‐year study period. LBP accounted for 1.3% of all ED presentations across the district. The keyword search did not identify any extra presentations. Total presentation numbers increased slightly each year: 4977 in 2015, 5074 in 2016, 5392 in 2017, 5414 in 2018 and 5652 in 2019 (compound annual growth rate = 3.2%).

### 3.2 Baseline characteristics

The mean age of patients was 49.2 years (SD 20.0), 51.7% were female, and 10.3% identified as Aboriginal or Torres Strait Islander (7.1% of residents in the health district identify as Aboriginal or Torres Strait Islander^30^). The majority of patients resided in major city areas (44.1%) or inner regional areas (38.5%) and were triaged as semi‐urgent (60.5%) and urgent (25.6%). They were primarily self‐referred (93.4%), with a small portion referred by a general practitioner (2.8%). 8.0% were re‐presentations to the ED within 5 days, and 18.4% were admitted to the hospital at the end of their ED visit (Table 2).

**TABLE 2:** Baseline characteristics and analysis by facility remoteness classification

Variable	Total sample (36 EDs)	Major cities (5 EDs)	Inner regional (12 EDs)	Outer regional (19 EDs)
Total presentations, % (*n*)	100% (26 509)	46.8% (12 399)	37.1% (9824)	16.2% (4286)
Age (years), mean (SD)	49.2 (20.0)	49.8 (20.3)	48.1 (19.7)	50.0 (19.4)
Gender (Female), % (*n*)	51.7% (13 701)	52.3% (6482)	51.2% (5030)	51.1% (2189)
Aboriginal and/or Torres Strait Islander, % (*n*)	10.3% (2738)	6.6% (824)	12.3% (1205)	16.5% (709)
Geographical location of patient's postcode, % (*n*)				
Major cities	44.1% (11 699)	88.5% (10 975)	6.0% (583)	3.3% (141)
Inner regional	38.5% (10 205)	10.3% (1276)	88.4% (8688)	5.6% (241)
Outer regional	16.7% (4433)	0.7% (81)	5.1% (500)	89.9% (3852)
Remote and very remote	0.2% (51)	<0.1% (8)	0.1% (11)	0.7% (32)
Triage code, % (*n*)				
Resuscitation	<0.1% (2)	<0.1% (2)	— (0)	— (0)
Emergency	4.4% (1164)	5.9% (736)	3.5% (341)	2.0% (87)
Urgent	25.6% (6775)	21.1% (2610)	26.0% (2552)	37.6% (1613)
Semi‐urgent	60.5% (16 045)	64.6% (8006)	60.1% (5902)	49.9% (2137)
Non‐urgent	9.5% (2522)	8.4% (1044)	10.5% (1029)	10.5% (449)
Presentation time, % (*n*)				
AM	45.8% (12 128)	46.0% (5700)	46.0% (4516)	44.6% (1912)
PM	54.2% (14 381)	54.0% (6699)	54.0% (5308)	55.4% (2374)
Within standard business hours (09:00–17:00)	53.0% (14 042)	53.1% (6578)	52.9% (5196)	52.9% (2268)
Outside standard business hours	47.0% (12 467)	46.9% (5821)	47.1% (4628)	47.1% (2018)
Referral source (top 2), % (*n*)				
Self, family, friends	93.4% (24 756)	91.9% (11 397)	94.4% (9269)	95.4% (4090)
General practitioner or dentist, not hospital‐based	2.8% (755)	4.4% (546)	1.5% (150)	1.4% (59)
Minutes in ED, median (IQR)	164 (92–255)	206 (132–305)	146 (84–231)	89 (49–158)
>240 min in ED, % (*n*)	27.1% (7182)	36.3% (4496)	22.2% (2184)	11.7% (502)
Cost per presentation (AUD$), median (IQR)	$444 ($410–$649)	$444 ($431–$678)	$444 ($408–$634)	$452 ($388–$643)
Re‐presentation within 5 days, % (*n*)	8.0% (2124)	6.0% (748)	8.8% (869)	11.8% (507)
Hospital admissions, % (*n*)	18.4% (4888)	20.4% (2525)	15.8% (1557)	18.8% (806)
Admission length of stay (days), median (IQR)	2 (1–5)	2 (1–5)	2 (1–5)	2 (1–5)
Cost per admission (AUD$), median (IQR)	$3214 ($1521–$6644)	$3299 ($1521–$7506)	$3089 ($1521–$5446)	$3336 ($1937–$5025)

### 3.3 Analysis by remoteness classification

Table 2 shows ED and related admission data by facility remoteness classification.^20^, ^21^ We did not include remote or very remote classifications as there are no facilities in the health district that are classified as this. Across the study period, there were 12 399 (46.8%) ED presentations in a major city area, 9824 (37.1%) in an inner regional area and 4286 (16.2%) in an outer regional area.

### 3.4 Analysis by NSW Health delineation level

Table 3 shows ED and related admission data by NSW Health delineation level.^28^, ^29^ There were 5128 presentations at the Level 6 facility, 5924 at the Level 5 facilities, 5691 at the Level 4 facilities, 6131 at the Level 3 facilities, 3220 at the Level 2 facilities and 415 at the Level 1 facilities. Median time in ED was longest in the Level 6 facility (230 min) and quickest in the Level 1 facilities (58 min).

**TABLE 3:** Analysis by NSW Health delineation level

Variable	6^a^ (1 ED)	5^a^ (2 EDs)	4^a^ (3 EDs)	3^a^ (8 EDs)	2^a^ (16 EDs)	1^a^ (6 EDs)
Total presentations, % (*n*)	19.3% (5128)	22.3% (5924)	21.5% (5691)	23.1% (6131)	12.1% (3220)	1.6% (415)
Age (years), mean (SD)	49.1 (20.8)	47.8 (19.5)	49.6 (20.1)	48.7 (19.6)	52.4 (19.5)	48.1 (19.7)
Gender (female), % (*n*)	52.7% (2704)	50.2% (2971)	52.1% (2966)	51.4% (3150)	52.2% (1680)	55.4% (230)
Aboriginal and/or Torres Strait Islander, % (*n*)	5.7% (293)	16.4% (836)	8.6% (492)	12.6% (770)	9.7% (312)	8.4% (35)
Minutes in ED, median (IQR)	230 (156–342)	165 (105–240)	186 (115–277)	128 (70–217)	93 (50–170)	58 (33–113)
>240 min in ED, % (*n*)	44.4% (2279)	24.7% (1465)	30.7% (1748)	19.7% (1209)	13.8% (444)	8.9% (37)
Cost per presentation (AUD$), median (IQR)	$491 ($438–$774)	$439 ($431–$600)	$467 ($438–$649)	$439 ($408–$621)	$556 ($408–$754)	$410 ($369–$620)
Re‐presentation within 5 days, % (*n*)	5.6% (286)	7.9% (469)	8.0% (457)	8.4% (514)	11.0% (353)	10.8% (45)
Hospital admissions, % (*n*)	30.4% (1558)	19.2% (1136)	9.8% (560)	13.5% (826)	24.1% (775)	8.0% (33)
Admission length of stay (days), median (IQR)	2 (1–5)	1 (1–3)	4 (2–7)	3 (1–6)	2 (1–5)	3 (1–5)
Cost per admission (AUD$), median (IQR)	$3089 ($1352–$7483)	$2407 ($885–$4295)	$4274 ($2569–$8866)	$3544 ($2274–$7060)	$3307 ($1843–$4303)	$3336 ($1642–$8356)

Note

Level 1 (7 facilities)—provides primary care assessment within designated area. 24‐h on‐site staff with basic life support (BLS), minimum level of service. Level 2 (16 facilities)—provides emergency care within designated area, BLS and advanced life support (ALS) should be available and may have access to allied health, and emergency caseload may be intermittent. Level 3 (8 facilities)—manages full range of emergency presentations, provides 24‐h triage service and provides primary emergency care, incl short‐term mechanical ventilation, allied health available, medical practitioner available 24 h. Level 4 (3 facilities)—Level 3 PLUS provides definitive care for most emergency presentations, incl invasive monitoring. Specialist emergency medicine staff on‐site, allied health available. Level 5 (2 facilities)—Level 4 PLUS ability to manage critically ill patients, speciality services on‐site for consultation, extended hour access to selected allied health (e.g. social worker and/or physiotherapist). Level 6 (1 facility)—Level 5 PLUS ability to manage all complex emergencies, referral centre for complex patients from lower level service, speciality services on‐site (e.g. neurosurgery, cardiothoracic surgery).

^a^ Description based on NSW Ministry of Health.^28^

### 3.5 Analysis by ED staffing portfolio

Table 4 shows ED and related admission data by the ED staffing portfolio. Most ED presentations were at facilities staffed by FACEM staff (18 563) compared with those staffed by GPVMO staff (7043). FACEM‐staffed facilities had a longer median time in ED (192 min) than GPVMO‐staffed facilities (94 min). Both staffing portfolios had the same admission rate into hospital (18.6%).

**TABLE 4:** Analysis by ED staffing portfolio

Variable	Fellowship of Australasian College of Emergency Medicine (FACEM) staff (7 EDs)	General Practitioner Visiting Medical Officer (GPVMO) (29 EDs)
Total presentations, % (*n*)	70.0% (18 563)	26.6% (7043)
Age (years), mean (SD)	49.4 (20.2)	49.1 (19.4)
Gender (female), % (*n*)	51.7% (9597)	52.0% (3659)
Aboriginal and/or Torres Strait Islander, % (*n*)	9.3% (1719)	13.4% (943)
Minutes in ED, median (IQR)	192 (122–287)	94 (52–167)
>240 min in ED, % (*n*)	32.9% (6108)	12.6% (890)
Cost per presentation (AUD$), median (IQR)	$444 ($431–$649)	$442 ($408–$643)
Re‐presentation within 5 days, % (*n*)	7.1% (1319)	10.3% (728)
Hospital admissions, % (*n*)	18.6% (3451)	18.6% (1312)
Admission length of stay (days), median (IQR)	2 (1–5)	2 (1–5)
Cost per admission (AUD$), median (IQR)	$3089 ($1340–$7122)	$3336 ($1989–$5031)

Note

NB: It does not include 903 presentations from the facility with a shared model of care. These unadjusted findings may be confounded by the ED setting/location.

## 4 DISCUSSION

Our study highlights key differences in ED service indicators with respect to presentations for LBP across remoteness areas and staffing portfolios in a large NSW Local Health District. The time patients spent in ED was shorter in outer regional EDs and EDs staffed by GPVMOs. Re‐presentation rates were higher in outer regional EDs and EDs staffed by GPVMOs than in major city EDs. We observed notable differences between delineation levels. For example, patients spent longer in EDs at Level 5 and 6 facilities than lower delineation levels (230 min versus 165 min, respectively), and re‐presentation rates were lower. Hospital admission rates were highest in the Level 6 facility and lowest in Level 1 facilities.

A strength of our study is we examined a large number of LBP presentations to EDs across a large number of facilities with varying remoteness areas, delineation levels and staffing portfolios. We have high confidence that we sampled all LBP presentations over the 5‐year study period as we used a number of data extraction mechanisms to filter these. The data quality was high, being based on clinical notes taken by ED staff at the point of care delivery, and then reviewed and entered by clinical data coders after patient discharge. We also extracted and report real costs for LBP ED presentations and admissions. A limitation is we may have missed some presentations where LBP was present but not the principal or secondary diagnosis. Our study also does not provide information about EDs in remote or very remote areas or any capital cities of Australia, as there are none within the sampled health district.

Data from our study align with previous ED presentation rates for LBP in Australia. We found a presentation rate within our sample of 1.3%, which is similar to the previously described rates, ranging from 1.9% to 3.4%.^8^, ^9^, ^31^ Another study of LBP presentations across EDs in NSW reported the highest presentation rates in rural areas based on age‐standardised rates within local government areas.^14^ However, our study used remoteness area and delineation levels based on the Australian Statistical Geography Standard Remoteness Structure^20^, ^21^ and found that LBP represented a smaller proportion of all ED presentations in regional and rural areas than in major city areas. The cost of an ED visit for LBP within our sample (median AUD$444 and mean AUD$547) was comparable to that found by Coombs, Machado^32^ for non‐ambulance presentations (mean AUD$584). However, the cost of an admission for LBP from ED was significantly lower in our sample (median AUD$3214 and mean AUD$5265) than in the previous study sample (mean AUD$13 137).^32^ This difference may be due to slightly different inclusion criteria between the studies; Coombs, Machado^33^ included non‐specific low back pain and lumbosacral radicular syndromes, while we only included non‐specific low back pain. No other studies have compared data on time in ED, re‐presentation rate, admissions, or cost across remoteness areas, staffing portfolio or delineation level.

The variation in ED presentations and performance measures observed in our study across ED types highlights the importance of considering local context when developing service improvement strategies. A performance measure for NSW EDs is achieving the emergency treatment performance target of 81% of all patients discharged from ED, admitted to hospital or transferred to another hospital within 4 h.^16^ Other indicators include, but are not limited to, a timely triage (<5 min) process and limited rates of re‐presentation.^16^ All EDs (irrelevant of remoteness location, delineation level or staffing portfolio) are currently measured against the same performance indicators. These facilities are, however, different in the way they function and the patients they see. Our study shows that hospitals with different staff and across different locations do not consistently meet the above targets. This means the general targets across sites might not be a good indicator of service performance, and any solution to improve such targets needs to be tailored for the service challenges.

When considering the health care of regional and rural areas, it is critical to consider Aboriginal and Torres Strait Islander communities.^34^ Higher ED attendance rates for LBP by Aboriginal and Torres Strait Islander patients in more regional/rural EDs may reflect higher populations in those areas.^35^ However, it may also be due to the fact that Aboriginal and Torres Strait Islanders are 1.2 times more likely to report having a back problem than other Australians.^36^ It may also be a combination of these two factors. While all EDs should ensure they are culturally safe, the numbers of presentations highlight the need to ensure that services in regional and rural areas have embedded culturally safe practices and environments to enable equitable health care for these communities.

Hospitals, including the ED, are complex systems made up of multiple interconnected parts (e.g. staffing levels, bed availability, presentation numbers).^37^ There are many reasons, internal and external to the ED, which may affect these different parts. While we have only described service indicators by three selected ED characteristics (remoteness level, delineation level and staffing portfolio), there are others that may need to be considered. These may include, but are not limited to, the support available within the community (e.g. GP appointment availability), the size of the hospital or ED, the presentation referral source (e.g. self versus GP), the mode of arrival into ED (e.g. ambulance) or the clinical presentation of the patient (e.g. duration of LBP, co‐morbidities). Although the complexity of hospital systems is well described, previous studies have only concentrated on describing LBP presentations to EDs within metropolitan areas^8^, ^9^ or only considered the geographical location of the ED.^14^ Due to the complexity of the ED environment, it is vital to consider other factors that may impact LBP presentations and the local context for each ED.

Currently, there is little research or health care policy focus on the prevalence and burden of LBP and other musculoskeletal conditions in regional and rural areas, even though our study shows that the service‐level indicators are clearly different across facilities and areas. Therefore, context‐specific research in these areas is required. Further research should describe LBP presentations across other rural and remote facilities and include information regarding treatments delivered, and patient outcome data, in EDs across locations. This information about local services will enable relevant and targeted strategies to be implemented to improve patient care. Service indicators and patient journey factors (e.g. time spent in ED) can directly impact patients and the quality of care received.^38^ The differences in health care provision in metropolitan, regional and rural areas are well documented; however, little is known about the impact of staffing portfolios on service‐level indicators. Our study identified differences in ‘time in ED’, ‘re‐presentation in 5 days’ and ‘cost per admission’ between staffing portfolios, but we do not know why these differences exist. The reasons for these differences across locations and staffing portfolios should be further investigated. This could involve qualitative exploration of clinician and patient experiences to better understand and manage these differences.

## 5 CONCLUSIONS

This study describes LBP presentations across 37 EDs and highlights the potential burden these presentations may place on hospitals. A higher proportion of ED patients in regional areas re‐present for LBP, and these patients also have shorter ED stays. In contrast, hospital admission rates appear variable across localities. LBP presentations appear to follow different pathways depending on the ED remoteness area, delineation level and staffing portfolio. Given the differences, any implementation or support mechanisms should consider local context.

## CONFLICT OF INTEREST

None.

## AUTHOR CONTRIBUTIONS

SRED: conceptualization; data curation; formal analysis; investigation; methodology; project administration; validation; visualization; writing – original draft; writing – review & editing. SJK: conceptualization; methodology; supervision; writing – review & editing. RH: conceptualization; data curation; formal analysis; methodology; supervision; writing – review & editing. MOF: conceptualization; writing – review & editing. KC: writing – review & editing. JPS: writing – review & editing. AT: writing – review & editing. JL: writing – review & editing. JB: writing – review & editing. MB: data curation; writing – review & editing. CMW: conceptualization; funding acquisition; methodology; project administration; supervision; writing – review & editing.

## DISCLOSURE

CW and SK received salary funding from the Australian National Health and Medical Research Council.

## ETHICS APPROVAL

HNELHD Human Research Ethics Committee granted ethics approval for this study (2019/ETH12178).

## Data Availability

Data used for analysis will be made available on reasonable request. Proposals for data use may be submitted to the principal/corresponding author.
